# *In vitro* topological loading of bacterial condensin MukB on DNA, preferentially single-stranded DNA rather than double-stranded DNA

**DOI:** 10.1038/srep29469

**Published:** 2016-07-08

**Authors:** Hironori Niki, Koichi Yano

**Affiliations:** 1 Microbial Genetics Laboratory, Genetic Strains Research Center, National Institute of Genetics, 1111, Yata, Mishima, Shizuoka, 411-8540 Japan; 2 Department of Genetics, SOKENDAI (The Graduate University for Advanced Studies) 1111, Yata, Mishima, Shizuoka, 411-8540 Japan

## Abstract

Condensin is the major driving force in the segregation of daughter chromosomes in prokaryotes. Core subunits of condensin belong to the SMC protein family, whose members are characterized by a unique ATPase activity and dimers with a V-shaped structure. The V-shaped dimers might close between head domains, forming a ring structure that can encircle DNA. Indeed, cohesin, which is a subfamily of SMC proteins, encircles double-stranded DNA to hold sister chromatids in eukaryotes. However, the question of whether or not condensin encircles the chromosomal DNA remains highly controversial. Here we report that MukB binds topologically to DNA *in vitro*, and this binding is preferentially single-stranded DNA (ssDNA) rather than double-stranded DNA. The binding of MukB to ssDNA does not require ATP. In fact, thermal energy enhances the binding. The non-SMC subunits MukF and MukE did stimulate the topological binding of MukB, although they hindered DNA-binding of MukB. Recent reports on the distribution of condensin in genomes reveal that actively transcribed genes in yeast and humans are enriched in condensin. In consideration of all these results, we propose that the binding specificity of condensin to chromosome is provided not by the DNA sequence but by the DNA structure, which is ssDNA.

Condensin is an important protein complex for the compaction of chromosomal DNA in organisms ranging from bacteria to humans^1^,^2^,^3^. Prokaryotic condensin consists of a homodimer of an SMC core unit and two types of non-SMC subunits. The SMC homodimer binds to double-stranded DNA, and to ssDNA^4^,^5^. The MukBEF complex is a bacterial condensin complex in *Eshcrichia coli* and related bacteria^6^. MukB is the SMC protein, and MukE and MukF are non-SMC subunits. MukF, which is a member of the kleisin protein family^7^, forms a stable complex with MukE *in vivo*^8^. In living cells that encode MukB-GFP, a portion of the MukB molecules are distributed to discrete foci in the vicinity of the origin of replication in collaboration with MukE and MukF^9^,^10^. Thus the MukBEF complexes are formed at specific sites on a bacterial chromosome, and then each of the MukBEF-DNA complexes assembles to pack chromosomal DNA into a nucleoid. However, the mechanism by which particular chromosome sites are chosen for loading of the MukBEF complex remains unclear. Strangely, an *in vitro* analysis indicated that the DNA-binding of MukB is remarkably inhibited by non-SMC subunits of MukE and MukF^8^. In an earlier study, a globular domain of MukB was shown to have DNA-binding ability^11^. And MukF is known to interact with the globular domains of MukB^12^, suggesting that MukF covers the DNA-binding domain of MukB. To address this conflict between the *in vivo* and *in vitro* results regarding the DNA-binding of MukB, we considered that a ring structure of MukB dimers holds DNA strands as seen in a cohesion ring. Although previous reports have suggested that condensin physically encircles DNA in an isolated condensin-DNA complex^13^,^14^, it remains a matter of controversy whether condensin binds topologically. In the case of cohesin, the topological binding has recently been established by a topological loading assay for purified yeast cohesion, which is developed by Murayama and Ullmann (2014) (*MU assay* hereinafter)^15^,^16^. In the present study, we investigated the topological binding of bacterial condensin to DNA by using the MU assay with some modifications.

## Results

### Assay for *in vitro* topological loading of MukB on DNA

**Figure 1:**
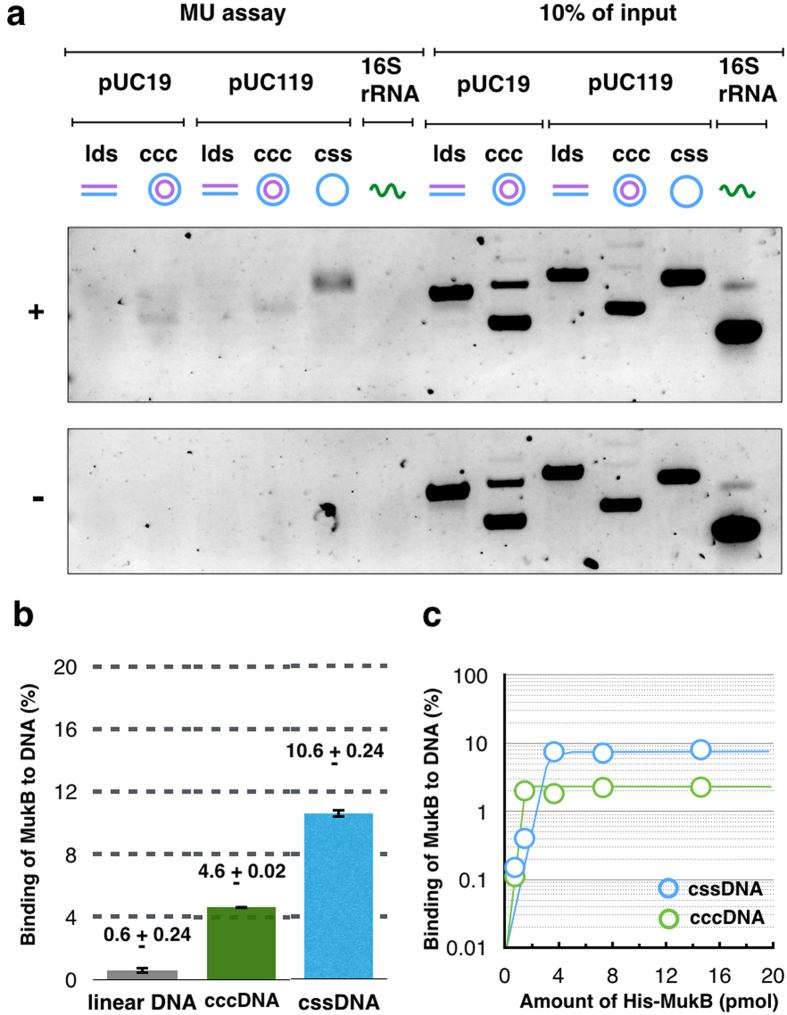
*In vitro* loading of MukB onto DNA. (**a**) Agarose gel electrophoresis of retrieved DNA from the MU assay. Various types of DNA and 16S rRNA were tested for the MU assay with histidine-tagged MukB (+) and without it (−). (**b**) Quantification of retrieved DNA by the MU assay. The means and standard deviation were calculated from two independent experiments. (**c**) Kinetics of histidine-tagged MukB loading onto cssDNA (blue) and cccDNA (green). Various amounts of histidine-tagged MukB were tested with pUC119 DNA (100 ng).

To test the topological loading of MukB on DNA, the MU assay was applied for purified MukB proteins. The hexa-histidine-tagged proteins of each of MukB, MukE, and MukF were considered biologically functional because they could complement their deletion phenotypes (data not shown), and then each protein was purified (Supplementary Fig. S1). Purified histidine-tagged MukB was incubated with a variety of DNA substrates under a condition of low salt (25 mM KCl), and then histidine-tagged MukB was recovered by affinity beads to a histidine-tag after washing with high salt buffer (750 mM KCl) to eliminate the salt-sensitive binding of MukB to DNA. DNA substrates that were pulled down with histidine-tagged MukB were analyzed by agarose gel electrophoresis. Linear double-stranded DNA (ldsDNA) was not retrieved, but both covalent closed circular DNA (cccDNA), which is dsDNA, and cssDNA were retrieved with histidine-tagged MukB (Fig 1a). The efficiency of DNA retrieval was 4.6% for cccDNA, and it was 10.6% for cssDNA (Fig 1b). The retrieval of circular DNA was significantly higher than the 0.6% retrieval observed for ldsDNA. MukB continuously preferred to bind to cssDNA rather than to other DNA substrates, although the ratios of retrieved DNA varied in each lot of the purified protein (Supplementary Fig. S2). Thus, MukB has the ability to bind to DNA in a salt-resistant manner. This binding is dependent on the topology of DNA substrates, suggesting that MukB topologically loads on DNA.

### Preferential loading of MukB to cssDNA

To confirm a preference for DNA-binding to circular DNA, we tested the binding kinetics of MukB to cccDNA and cssDNA, respectively. When cssDNA was used as the substrate, a higher yield of retrieved DNA was obtained (Fig 1c). Thus the MU assays indicate that MukB has a DNA-binding activity that is resistant to high salt, and it prefers cssDNA to ldsDNA and cccDNA for DNA-binding.

**Figure 2:**
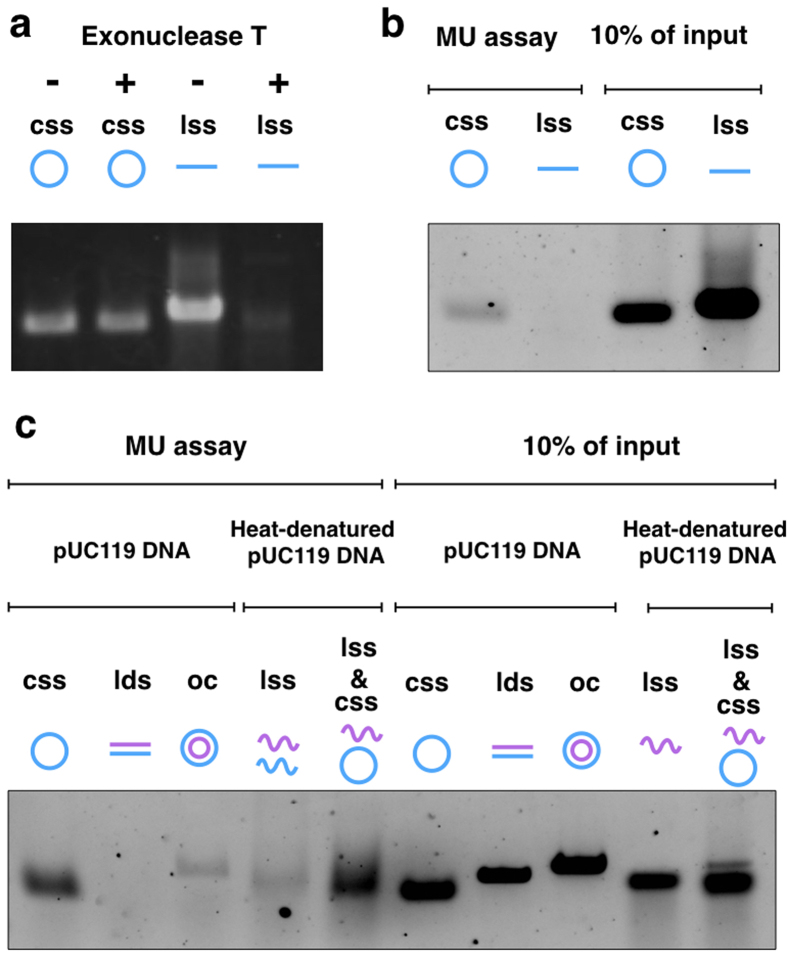
DNA Topology and single strand-mediated DNA-binding of MukB. (**a**) Agarose gel electrophoresis of ssDNA. Exonuclease T completely digested lssDNA of pUC119 (184 ng), but not cssDNA (100 ng). The gel was stained by ethidium bromide. (**b**) Agarose gel electrophoresis of retrieved ssDNA from the MU assay. The assay used 100 ng of ssDNA in each reaction. (**c**) Agarose gel electrophoresis of the MU assay using heat-denatured DNA. ldsDNA and ocDNA were boiled at 95 °C for 5 min, and then quickly chilled.

It was uncertain whether MukB preferentially bound to ssDNA independent of the DNA topology. We tested the binding of MukB to linear single-stranded DNA (lssDNA). To prepare lssDNA, cssDNA of pUC119 was hybridized with a DNA oligomer including the *Pst*I site, and then cut by the restriction enzyme *Pst*I. All of the hybridized cssDNAs were almost certainly linearized because they were sensitive to exonuclease T, which is a ssDNA-specific nuclease (Fig. 2a). No lssDNA was retrieved by the MU assay (Fig. 2a). In addition, we used heat-denatured DNAs for the MU assay. Heating of ldsDNA produces lssDNA, and heating of open circular DNA (ocDNA) produces both lssDNA and cssDNA. Heat-treated ocDNA became a more effective substrate for DNA-binding of MukB, but heat-treated ldsDNA did not (Fig. 2c). Thus, efficient retrieval of the DNA substrate by using the MU assay was required for not only ssDNA, but also DNA topology. These results indicate that MukB holds onto DNA within its ring structure—i.e., topological binding occurs in the case of cohesin.

### Effect of SSB on the topological binding of MukB

**Figure 3:**
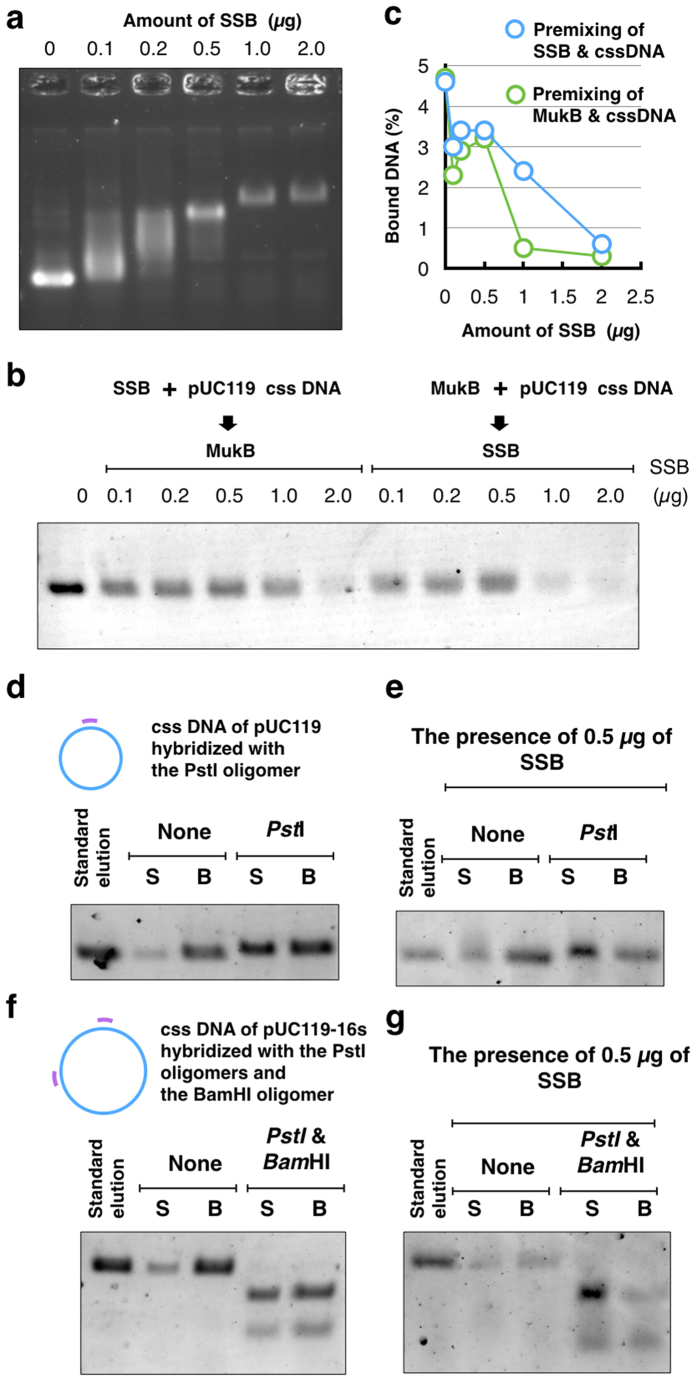
SSB and Topological binding of MukB. (**a**) Agarose gel electrophoresis of gel mobility shift assay. Various amounts of *E. coli* SSB were bound to the cssDNA of pUC119 (100 ng) after incubation in HKD buffer on ice for 10 min. (**b**) Agarose gel electrophoresis of the MU assay in the presence of SSB. Pre-incubation of SSB and cssDNA was carried out as follows. A mixture of SSB and cssDNA was incubated on ice for 10 min, and then histidine-tagged MukB (7.3 pmol) was added. Next, the mixture was incubated on ice for 5 min, and then incubated at 37 °C for 10 min before the MU assay. On the other hand, pre-incubation of histidine-tagged MukB and cssDNA was carried out as follows. A mixture of MukB and cssDNA was incubated on ice for 10 min, and then SSB was added. Next, the mixture was incubated on ice for 5 min, and then it was incubated at 37 °C for 10 min before the MU assay. (**c**) Quantification of retrieved DNA from the MU assay in the presence of SSB. Premixing of SSB and cssDNA is indicated in blue circles, and premixing of MukB and cssDNA is indicated in green circles. (**d**) The cssDNA hybridized with the oligomer including a *Pst*I site was analyzed by the MU assay. After washing the immobilized MukB-DNA complexes on beads, they were digested with the restriction enzyme *Pst*I, and the reaction mixture was separated into a supernatant fraction (S) and a bead fraction (B). (**e**) SSB (0.5 μg) was added to the digestion reaction. (**f**) The cssDNA hybridized with the oligomers including the *Pst*I site or the *Bam*HI site was analyzed by the MU assay. The immobilized MukB-DNA complex on beads was digested with the restriction enzymes *Pst*I and *Bam*HI. (**g**) SSB (0.5 μg) was added to the digestion reaction.

*In vivo*, single-stranded-binding proteins (SSBs) protect ssDNA from attack by DNA-damage agents or remove the secondary structure of DNA. In *E. coli*, tetramers of SSB are strongly bound to ssDNA of the chromosome so that other DNA-binding proteins cannot access the ssDNA segment covered by SSB. We examined the effects of SSB on the binding of MukB to cssDNA. As the concentration of SSB was increased, the mobility of cssDNA was proportionally shifted in an agarose gel and the saturation binding of SSB was 2.0 μg in a reaction mixture (Fig. 3a). The saturation binding of SSB to cssDNA markedly inhibited the topological binding of MukB to cssDNA (Fig. 3b). Pre-mixing of cssDNA and MukB on ice did not improve the efficiencies of cssDNA retrieval by MukB. These results suggest that a direct interaction of MukB with ssDNA might be essential for topological binding of MukB to cssDNA.

### Release of MukB from cssDNA by linearization

When the ring of SMC dimers entraps circular DNA, linearization of the circular DNA causes the entrapped DNA to slide out from the SMC ring^15^. To linearize cssDNA that was bound by MukB, cssDNA was previously hybridized with oligomers that included a recognition site by a restriction enzyme. In the MU assay using hybridized cssDNA, the retrieved DNA on beads was digested by *Pst*I (Fig. 3d) or both *Pst*I and *Bam*HI (Fig. 3f). A large amount of cssDNA was released into the supernatant of a DNA-loading reaction after digestion, although a small amount of cssDNA was recovered in the supernatant without digestion. Even though cssDNA was completely digested by both *Pst*I and *Bam*HI, a large amount of cssDNA remained on the beads (Fig. 3f). It was likely that the secondary structure of ssDNA hindered the release of digested DNA from the ring of MukB. To improve the release of DNA from the MukB ring, the secondary structure of ssDNA was removed by SSB. Addition of a moderate amount of SSB (0.5 μg) enhanced the release of the digested DNA from the MukB immobilized on beads, as expected (Fig. 3e,g). These results support the notion that the MukB condensin entraps ssDNA.

### Effect of MukE and MukF on topological binding of MukB

**Figure 4:**
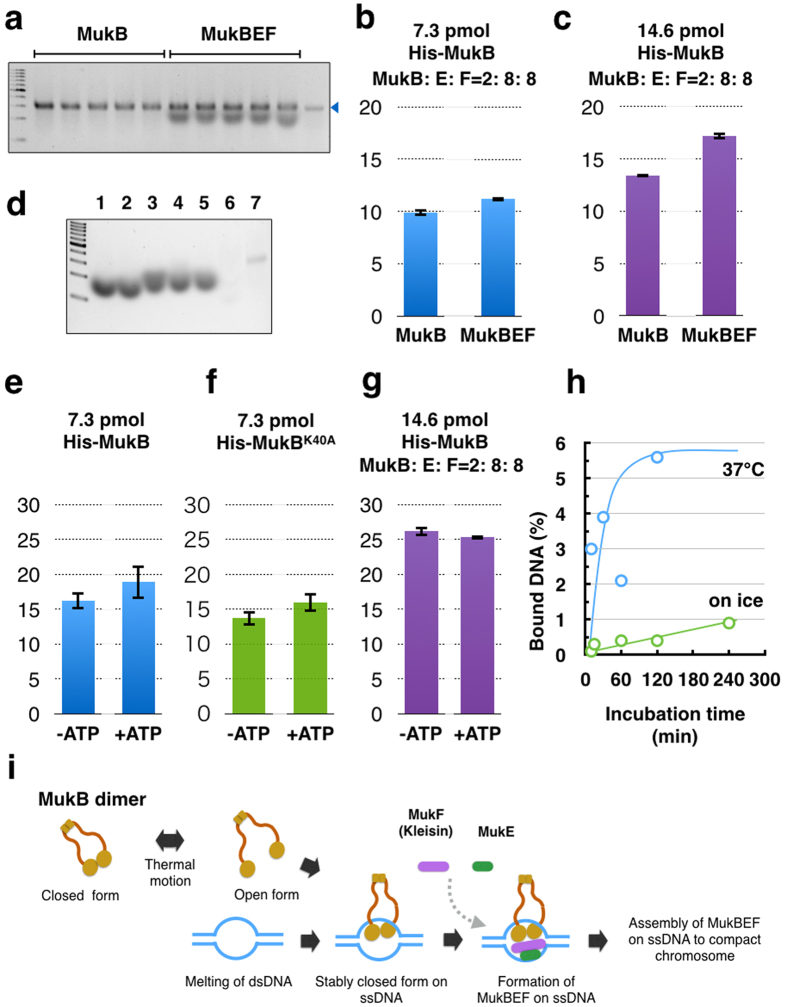
Topological DNA-binding of histidine-tagged MukB in the presence of MukEF and ATP. (**a**) Agarose gel electrophoresis of the MU assay. Five reactions with MukB or MukBEF were analyzed and detected by fluorescence of SYBRGreen II. The arrowhead indicates cssDNA of pUC119. (**b**) Effect of non-SMC subunits, MukE and MukF on retrieved DNA from the MU assay; MukB (7.3 pmol), MukE (29.2 pmol), MukF (29.2 pmol), and pUC119 cssDNA (100 ng). Means and standard deviations were calculated from five independent experiments. (**c**) Effect of non-SMC subunits, MukE and MukF on retrieved DNA from the MU assay; MukB (14.6 pmol), MukE (58.4 pmol), MukF (58.4 pmol), and pUC119 cssDNA (100 ng). Means and standard deviations were calculated from five independent experiments. (**d**) Agarose gel electrophoresis of MukF alone. Purified histidine-tagged MukF in HKD buffer was loaded in agarose gel electrophoresis and stained by SYBRGreen II. The results are shown for MukF (30 ng) incubated at 37 °C overnight (lane 1), MukF without incubation (lane 2), MukF incubated with DNase I at 37 °C overnight (lane 3), MukF incubated with RNase A at 37 °C overnight (lane 4), MukF incubated with mung bean nuclease at 37 °C overnight (lane 5), MukF incubated with proteinase K at 37 °C overnight (lane 6), and 10 ng of cssDNA of pUC119 (lane 7). (**e**) Effect of 2 mM ATP on DNA retrieval from the MU assay. (**f**) Effect of 2 mM ATP on DNA retrieval of MukB^K40A^ from the MU assay. (**g**) Effect of 2 mM ATP on DNA retrieval from the MU assay; MukB (14.6 pmol), MukE (58.4 pmol), MukF (58.4 pmol), and pUC119 cssDNA (100 ng). Means and standard deviations were calculated from five independent experiments. (**h**) Effect of the reaction temperature on DNA retrieval from the MU assay. Reaction mixtures of MukB (7.3 pmol) and pUC119 cssDNA (100 ng) were incubated at 37 °C (blue) and on ice (green). The means and standard deviations (**b**,**d**,**e**) were calculated from five independent experiments. (**i**) A model of topological binding of MukB in *E. coli* cells.

To confirm the effect of non-SMC subunits in the topological binding of MukB, purified MukE and MukF were added to the reaction mixture of MukB and cssDNA. The stoichiometry of the MukBEF complex *in vivo* is estimated at B_4_E_4_F_2_ or B_2_E_4_F_2_, based on the quantitative analyses of fluorescent-labeled Muk proteins in a living cell^10^. On the other hand, biochemical analysis has suggested that the stoichiometry of active MukBEF is B_2_E_2_F_1_, and the stoichiometry of the inactive form that does not bind DNA is B_2_E_4_F_2_^8^. In the present study, MukE and MukF did not deteriorate topological binding of MukB when excess amounts of MukE and MukF (B_2_E_8_F_8_) were added to the reaction mixtures for the MU assay (Fig. 4a,b). On the contrary, the topological binding was significantly improved by the reaction with a higher concentration of proteins (Fig. 4c). The effect of MukE and MukF on topological binding of MukB is notable because these proteins deteriorate DNA-binding activity of MukB^8^. This result suggests that there are at least two modes of DNA binding of MukB to DNA *in vitro*.

When the cssDNA retrieved from the reaction with MukE and MukF was analyzed by agarose gel electrophoresis using the fluorescent dye SYBRGreenII (SGII), additional bands appeared below the band of cssDNA (Fig. 4a). These bands were broader than the band of cssDNA. The bands appeared in lanes on which MukF alone was loaded (Fig. 4d). Indeed, the emission of light by SGII was detected in a solution when MukF was mixed with SGII (Supplementary Fig. S3a). The band was sensitive to proteinase K, but not to nucleases. A profile of the absorbance spectrum showed that MukF had strong absorbance at 280 nm, but not 260 nm, which is the wavelength for the maximum absorbance of nucleic acids (Supplementary Fig. S3b). These results indicate that the MukF protein itself was detected in the agarose gel by SGII. This ability of MukF to bind to and activate SGII warrants further analysis in future studies.

### ATP is not required for topological binding of MukB

The MU assays above were carried out without ATP, indicating that topological binding of MukB to cssDNA does not require ATP. MukB is the ABC type of ATPase. Its ATPase activity is essential for physiological functions in *E. coli*. We tested the effect of ATP on binding of MukB to DNA. When 2 mM ATP was added to the reaction mixture, the amount of cssDNA retrieved was not significantly higher than in the reaction without ATP (Fig. 4e). To confirm the requirement of ATP hydrolysis, a mutant form of MukB was used for the reaction. The MukB^K40A^ protein has a mutation in an active center of ATP hydrolysis and cannot complement the deficiency of the MukB deletion mutation. However, efficient typological binding of MukB^K40A^ was observed (Fig. 4f). In addition, when 14.6 pmol of MukB with MukE and MukF (B_2_E_8_F_8_) was incubated with cssDNA, ATP addition did not stimulate the topological binding of MukB (Fig. 4g). Thus, energy transfer from ATP is not necessary for topological binding of the MukB to cssDNA. These results are consistent with previous reports that ATP hydrolysis enhances the dissociation of MukBEF from DNA, but not the association of MukBEF to DNA^10^,^17^. Instead, the temperature after mixing cssDNA with MukB is critical for the efficient binding of MukB. The binding reaction was not boosted on ice, but was quickly enhanced at 37 °C (Fig. 4h). Thus thermal motion is an important promoter of the formation of the topologically bound MukB-DNA complex.

## Discussion

We identified topological loading of MukB to circular DNA, although the ratio of topological binding on DNA was low (Fig. 1b). In the case of the fission yeast cohesin, about 5% of input DNA was retrieved in the presence of cohesin alone, although the Mis4-Ssl3 cohesin loader improved the amount of retrieved DNA to about 25%, depending on the presence of ATP^15^. It has been considered that there is no loader for the MukB condensin in *E. coli*, based on the phenotypes of gene deletion mutants in a gene knockout library of *E. coli*^18^.

Although the suggested preference for ssDNA over dsDNA is apparent in Fig. 1b,c, it should be noted that this preference is not so pronounced at lower concentrations of purified MukB. Thus, we cannot exclude the possibility that MukB exhibits significant loading onto both dsDNA and ssDNA *in vivo*. However, the stable ssDNA segment that is produced by the opening of dsDNA is not ubiquitous on the bacterial genome, or rather it is restricted as chromosomal segments that were highly transcribed in the manner of rRNA and tRNA genes. Therefore, such restriction would contribute to the specific localization of the MukBEF complex in the vicinity of replication origin, as discussed below.

In contrast to the proportional binding of SSB to cssDNA, the ratio of retrieved cssDNA did not decrease in proportion to the amount of SSB (Fig. 3c). A moderate amount of SSB (0.2–0.5 μg) did not interfere with the binding of histidine-tagged MukB to cssDNA. Because SSB removes the secondary structure of cssDNA, cssDNA may be a better substrate for topological binding of MukB despite the masking of cssDNA by SSB. *In vivo*, free ssDNA is rapidly bound by SSB to protect the DNA region. As a matter of course, the complete covering by SSB inhibits access to the ssDNA. If moderate binding of SSB occurs only at a specific region on a bacterial chromosome, this would be helpful for the localization of MukB *in vivo*.

The condensin complexes that topologically bind to DNA were isolated from *Saccharomyces cerevisiae* and *Bacillus subtilis*^13^,^14^. Proteolytic cleavage of non-SMC subunits causes the release of DNA from the isolated complexes, indicating that the ring structure composed of the SMC core unit and non-SMC subunits is responsible for the topological engagement of DNA. By contrast, our reconstitution experiments of the MukB-DNA complex show that MukB alone is sufficient for the topological binding to DNA. We cannot exclude the possibility that this binding mode of MukB is specific *in vitro*. Alternatively, it is conceivable that the topological binding is the first step toward formation of the condensin-DNA complex, after which a more stable complex would be formed with non-SMC subunits. Interestingly, *E. coli* RecN, which is a highly conserved, DNA damage-inducible SMC protein, wraps DNA by forming a polymer via head-head engagement^19^. A similar binding mode of MukB could be included in the processes of condensin complex formation.

MukB^K40A^ could bind to DNA *in vitro* topologically (Fig. 4f). In contrast, many MukB mutants of ATPase activity have been analyzed and found to be incapable of binding to DNA *in vivo*^10^,^20^. However, the ATPase activity of MukB is not directly involved in DNA binding, but rather is involved in coordinating MukB interactions with non-SMA subunits and DNA^12^,^17^. In addition, the DNA binding of RecN is not dependent on its ATPase activity, because the ATPase-deficient RecN^K35A^ mutant can assemble at a damaged site on the chromosome^21^. Once RecN^K35A^ assembles, however, disassembly of RecN^K35A^ does not occur after the release of DNA damage stress, and thus the cell growth is inhibited. Therefore, it is likely that ATPase activity is not necessary for the binding of bacterial SMC proteins to DNA. Instead, ATPase activity would contribute to the turnover of assembled SMC proteins on DNA.

Electron microscopic observation has revealed two forms of the purified MukB: the open form is a V-shaped dimer, and the closed form is a filamentous dimer^22^,^23^. We hypothesize that the arms of MukB dimers are flexible and change between the open form or closed form depending on the thermal fluctuation (Fig. 4i). Occasionally the open form captures DNA, and then changes into the closed form. After the MukB dimer captures ssDNA, the closed form would become static because a part of the inner interface of a MukB globular domain interacts with ssDNA. Thus MukB would keep the captured DNA steady inside the ring of the dimer. Further, the MukB-DNA complex might be strengthened by MukEF, and then each of the MukBEF-DNA complexes would be assembled to compact chromosomal DNA. We infer that ATP hydrolysis is required for dissociation of the MukBEF-DNA complex from the assembled complexes.

As is usual with *in vitro* analysis, the biochemical properties of purified Muk proteins do not always reflect physiological functions faithfully. Even if the topological binding of MukB to DNA is functional *in vivo*, it is unclear whether the *in vivo* substrate is ssDNA or dsDNA. Nevertheless, our results motivate us to consider the topological loading of MukB to ssDNA *in vivo* because of an understanding about the restricted loading of condensin on chromosome. Recent reports have demonstrated that condensin is specifically localized at ssDNA segments in a cell. The binding of condensin to ssDNA is found in both *Schizosaccharomyces pome* and *Bacillus subtilis*^5^,^24^. Because SMC proteins maintain biochemical activities beyond the three domains of life, the property of topological binding of MukB to ssDNA may also apply to these SMC proteins. From this viewpoint, we could reconsider some of the previous results on the distribution of condensin in genomic DNA. First, the SMC proteins of *B. subtilis* are highly accumulated at rRNA genes and the tRNA genes, which are highly transcribed genes, in addition to the ParB-binding sites that are putative loading sites for condensin^25^. Moreover, rRNA genes of yeast also are rich in condensin^26^,^27^. In fact, a plasmid containing the rRNA gene has been isolated with the condensin complex from yeast, and this complex is also known to bind the plasmid DNA topologically^28^. Moreover, tRNA genes also are targets for the binding of yeast condensin^29^, and the clustering of tRNA genes that scatter at budding yeast chromosomes is governed by condensin^30^. Finally, two recent reports employing Chip-Seq analyses indicated that condensin is enriched at actively transcribed genes by RNA polymerase II in yeast and humans^31^,^32^. Condensin accumulates at unwound DNA at the transcription start site. These reports are well consistent with our present results, indicating that the specific loading of MukB to ssDNA can account for the *in vivo* localization of condensin at actively transcribed genes. It is thought that ssDNA is remarkably transient *in vivo*, and therefore only highly actively transcribed genes including the rRNA and the tRNA genes might be favorable target sites for topological loading by condensin. Indeed, multiple copies of the rRNA genes in *E. coli* and *B. subtilis* are dispersed in the vicinity of the origins of replication. This arrangement of the rRNA genes is suitable for packaging of newly replicated DNA strands in an orderly manner. Moreover, the real copy number of the rRNA genes is greater than that required for sufficient synthesis of rRNA^33^. This fact suggests that the multiple copies of the rRNA genes perform other functions in addition to their role in rRNA synthesis. In conclusion, the condensin-loading sites are determined by DNA states, rather than DNA sequences. Our findings suggest that ssDNA generated by active transcription is a more important site for biological activity than previously thought.

## Materials and Methods

### Preparation of substrate DNA

We used purified DNA of pUC19, pUC119, and pUC119-16S as substrates of the MU assay. The plasmid pUC119 is a derivative from pUC19 to produce ssDNA that was packaged into M13 phage particles. The DNA fragments encoding 16S ribosomal RNA were amplified from *E. coli* MG1655 by using a primer set: rrnB-16S-F(Bam) for ggaggatccaaattgaagagtttgatcatggctc, and rrnB-16S-R(Pst) for ctgctgcagtaaggaggtgatccaaccgc. The amplified DNA fragment was digested by *Bam*HI and *Pst*I, and then inserted into the *Bam*HI and *Pst*I sites of pUC119, resulting in pUC119-16S.

To extract ssDNA of pUC119 from M13 phage particles, *E. coli* MV1184 cells harboring pUC119 were cultured in 2 x YT medium (Bacto tryptone 16 g l^−1^, yeast extract 10 g l^−1^, NaCl 10 g l^−1^, pH 7.6) at 37 °C overnight. To package the ssDNA of pUC119 into M13 phage particles, the cells were infected with a helper page M13KO7. Phage lysate of M13KO7 was added into 1 ml of overnight culture of MV1184 harboring pUC119 under the condition that the multiplicity of infection (moi) was between 2 and 10. Infected cells were incubated at 37 °C for 1 hour with gentle shaking. The culture was placed into 10 ml of 2 x YT medium containing 50 μg ml^−1^ of kanamycin, and then it was incubated until full growth of cells. The culture was centrifuged to remove cells, and then the supernatant was further centrifuged to precipitate phage particles (100,000 rpm for 30 min using a TLS-110 rotor; model: Optima TLX, Beckman Coulter Inc.). The precipitate was resuspended in 100 μl of TE solution (10 mM Tris-HCl: pH 8.0, 0.1 mM EDTA) containing RNase A (5 μg ml^−1^) and DNase I (10 μg ml^−1^), and the suspension was incubated at 37 °C for 30 min. The pUC119 cssDNA was extracted with a saturated solution of phenol/chloroform. Finally, the extracted DNA was precipitated by ethanol precipitation and resuspended in TE buffer.

The pUC19 and 119 cccDNA were extracted from *E. coli* cells by using silica-membrane technology and further purified by using CsCl density-gradient ultracentrifugation (rotor: TLS-110; model: Optima TLX, Beckman Coulter Inc.). ocDNA was purified by density-gradient ultracentrifugation from a DNA solution of pUC119 that was kept at 4 °C in long-term storage as a laboratory stock.

lssDNA was made by cutting of cssDNA of pUC119. An oligomer including the *Pst*I site (#881; agtcgacctgcaggcatgca) was hybridized with cssDNA of pUC119 to generate a dsDNA segment. The mixture of the oligomer and pUC119 cssDNA in HKD buffer (25 mM HEPES-KOH; pH7.6, 25 mM KCl, 1 mM DTT) was heated at 65 °C for 10 min and gradually cooled down until room temperature. The hybridized DNA was digested by *Pst*I, and then purified by phenol/chloroform extraction. To test linearization of pUC119 cssDNA, digested DNA was analyzed by ExoT nuclease.

Ribosomal RNA was extracted form of *E. coli* cells by Phenol extraction. To isolate 16S RNA, crude RNA extract was centrifuged by 15–60% glycerol density gradient centrifugation (rotor: TLS-55; model: Optima TLX, Beckman Coulter Inc.).

*E. coli* strains and plasmid DNA were obtained from National Bioresource Center; E. coli, Mishima, Japan.

### Expression and purification of the Muk proteins

Each of the DNA fragments containing the *mukB*, the *mukE*, and the *mukF* gene was amplified by polymerase chain reaction (PCR) and cloned into an expression vector, pET-28a. The primers used for the PCR were as follows: a set of primers for *mukB* (mukB-F[Nde]; atcatcatatgattgaacgcggtaaatttcg, mukB-R[EcoBam]; GGAGGATCCGAATTCgctgccgccttaattttaact), a set of primers for *mukE* (mukE-F[Nde]; atcatcatatgtcatcgacaaatattgaacaag, mukE-R[Bam]; GGAGGATCCttattcttcctctccgctatc), and a set of primers for *mukF* (mukF-F; agtgaattttcccagacagtc, mukF-R[Eag]; TTTCGGCCGaatatttgtcgatgacatgcgc). The capital letters in the upper primer sequences indicate the DNA sequences that were inside each of the coding genes. The amplified DNA fragments including the *mukB*, and the *mukE* gene are inserted into the *Nde*I and the *Bam*HI site of pET-28a. Each of gene products tagged with 6 residues of histidines at their N-terminuses was able to suppress growth deficiency of the *mukB* and the *mukE* deletion mutant, respectively. On the other hand, the MukF protein was tagged with 6 residues of histidines at their C-terminuses because the MukF protein tagged with histidines at their N-terminus did not suppress a defect of the *mukF* deletion mutant. To construct such a *mukF* gene, the amplified DNA fragment including the *mukF* gene was digested by the *Nde*I. A protruding 5’ end of the DNA fragment was converted into a blunt end by using T7 DNA polymerase and further digested by *Eag*I. T4 polynucleotide kinase was used to add a phosphate to the ends of the DNA fragments. The modified DNA fragment was inserted into the *Nde*I and the *Eag*I site of pET-28a. The MukF tagged with 6 residues of histidines at their C-terminuses was able to suppress a defect of the *mukE* deletion mutant. All *muk* genes cloned into pET-28a were thoroughly sequenced to confirm the coding products tagged with (His)6. Each of the plasmids that expressed histidine-tagged Muk proteins was transformed into the host BL21(DE3).

To purify the histidine-tagged Muk proteins, BL21(DE3) harboring pET28a-mukB, pET28a-mukE or pET28a-mukF was inoculated into 20 ml of the L medium (Bacto tryptone 10 g l^−1^, yeast extract 5 g l^−1^, NaCl 5 g l^−1^, pH 7.4) with 15 μg ml^−1^ of kanamycin, and incubated at 37 °C for overnight. All the overnight cultures were inoculated into 1000 ml of L medium with 15 μg ml^−1^ of kanamycin and gentle shaking at 37 °C. When the optical density of absorbance 600 nm against in the culture reached 0.3, isopropyl β-D-1-thiogalactopyranoside (IPTG, final concentration: 0.1 mM) was added to the culture to induce histidine-tagged proteins. After further gentle shaking at 37 °C for 2 hours, cells in the culture were harvested by centrifugation, and then washed by HK buffer solution (50 mM HEPES-KOH: pH7.6, 100 mM KCl). Precipitated cells were frozen by liquid nitrogen and kept in a freezer at −80 °C. The deep freezer preserved the frozen cells for at least three months.

To extract the his-tagged Muk proteins, the frozen cells were thawed on ice with 30 ml of the sonication buffer (50 mM HEPES-KOH, pH 7.6, 100 mM KCl, 1 mM phenylmethylsulfonyl fluoride). Addition of lysozyme was prohibited because of the increment of contaminated proteins in the final fraction. The cell suspension on an ice bath was sonicated by using a BRNSON SONIFIER 250 (duty cycle, constant; output control, 2.5, 10 second, 15 times). Sonicated cells were centrifuged by 10,000 g for 10 min to remove the cell debris. The supernatant was decanted into a glass flask. To precipitate proteins in the supernatant, 8.8 g of ammonium sulfate with fine powder (30%) was carefully added to 50 ml of the supernatant fluid. After ammonium sulfate was dissolved thoroughly, the solution was stood on ice for 15 min. The mixture was then centrifuged (9,100 *g*, 10 min, 4 °C). For purification of the his-tagged MukE protein, ammonium sulfate precipitation at 50% salt saturation followed by 30% salt saturation; 6.3 g of ammonium sulfate was dissolved into the supernatant of ammonium sulfate precipitation at 30% salt saturation. After standing on ice for 15 min, the mixture was centrifuged again (9,100 *g*, 10 min, 4 °C).

The supernatant of ammonium sulfate precipitation was discarded and the precipitate was resuspended in 10 ml of equilibrium buffer (50 mM HEPES-KOH, pH 7.6, 100 mM KCl, 20 mM imidazole). To remove insoluble particles, the resuspended solution was further centrifuged at 9,100 *g* for 10 min. The resuspended solution was then applied into an open column containing 8 ml of TALON metal affinity resin (Clontech Laboratories Inc.) that was equilibrated against the equilibration buffer. The resin was washed with 15 ml of the wash buffer (30 mM HEPES-KOH, pH 7.6, 750 mM KCl, 10 mM imidazole). The histidine-tagged proteins were eluted from the resin by elution buffer (50 mM HEPES-KOH, pH 7.6, 100 mM KCl, 500 mM imidazole). Each 1 ml of the flow-through fraction was collected into micro-test tubes. To detect eluted protein, the absorbance at 280 nm in each fraction was measured, and then the peak fractions were pooled.

The fractions containing histidine-tagged protein were loaded onto a Resource Q column (1 ml; GE Healthcare) that was equilibrated against buffer A (50 mM HEPES-KOH, pH 7.6, 100 mM KCl). After loading of histidine-tagged protein, the column was washed with 25 ml of buffer A. The column was developed with a linear gradient between 100–1000 mM KCl by using 20 ml of buffer B (50 mM HEPES-KOH, pH 7.6, 1000 mM KCl). Each 1 ml of the flow-through fraction was collected into micro-test tubes. Fractions including histidine-tagged protein were dialyzed against buffer C (50 mM HEPES-KOH, pH 7.6, 100 mM KCl, 0.1 mM DTT, 50% (v/v) glycerol). The concentration of purified protein was measured by a method based on Bradford protein assay (Bio-Rad Protein Assay, Bio-Rad Laboratories). Bovine serum albumin was used as a standard to calculate the protein concentration.

### *In vitro* MukB-loading assay

We used a topological loading assay developed by Murayama and Ullmann (2014)^15^. The method was modified and adapted for our experimental purpose. In general, the loading assay of MukB on DNA was carried out in a 10 μl reaction mixture (25 mM HEPES-KOH, pH 7.6, 25 mM KCl, 1 mM DTT, 1 mM MgCl_2_, 100 ng of substrate DNA, and 7.3 pmol of his-tagged MukB). The reaction mixtures of the MU assay were prepared on ice, and then put into an incubator at 37 °C. After 10 min, the reaction tubes were transferred onto ice and supplemented with 500 μl of CP buffer (25 mM HEPES-KOH, pH 7.5, 500 mM KCl, 1 mM DTT, 1 mM MgCl_2_, 5% glycerol, 0.35% Triton X-100, 5 mM imidazole). In addition, 10 μl of slurry of Dynabeads His-Tag (Novex) that was equilibrated against CP buffer was added into the reaction tubes and gently mixed. The tubes were then gently rotated at 4 °C for 30 min. The magnetic beads were washed using CW1 buffer (25 mM HEPES-KOH, pH 7.5, 750 mM KCl, 1 mM DTT, 1 mM MgCl_2_, 0.35% Triton X-100, 5 mM imidazole) with rotating at 4 °C for 10 min. The magnetic beads were washed three times using CW1 buffer, followed by one time with CW2 buffer (25 mM HEPES-KOH, pH 7.5, 100 mM KCl, 1 mM DTT, 1 mM MgCl_2_, 0.1% Triton X-100, 5 mM imidazole). After the complete removal of CW2 buffer, 10 μl of elution buffer (25 mM HEPES-KOH, pH 7.5, 100 mM KCl, 1 mM DTT, 1 mM MgCl_2_, 500 mM imidazole) was added to each reaction tube and incubated at room temperature for 10 min. A supernatant of each reaction was transferred to a new tube and added to 2 μl of denature solution (8% sodium dodecyl sulfate, 0.1 M EDTA) and 3 μl of loading buffer (0.9% SDS, 50% glycerol, 0.05% bromophenol blue). These samples were analyzed by 0.8% agarose gel electrophoresis in 1 x TAE buffer (100 V, 30 min). The agarose gels were stained by SYBRGreen II (Takara Bio Inc.). Images of gels were captured by using LuminoGraph (ATTO) or LAS-4000 mini (Fujifilim), and the band intensities were analyzed by using ImageJ 1.48v.

In order to linearize cssDNA that was bound to histidine-tagged MukB, cssDNA that hybridized with oligomers including the *Pst*I site (#881) was used for the MU assay. For double digestion by *Pst*I and *Bam*HI, pUC119-16S DNA was cloned the 16S ribosomal DNA fragment into the *Pst*I and *Bam*HI site on the pUC119. Oligomers including the *Pst*I and the *Bam*HI site were hybridized with cssDNA of pUC119-16S (PstI #891; ctccttaCTGCAGgcatgca, BamHI #896;tacccggGGATCCcaaattga). To digest the retained cssDNA by restriction enzymes, after washing with CW2 buffer, the magnetic beads were resuspended in 10 μl of digestion buffer (25 mM HEPES-KOH, pH 7.5, 750 mM KCl, 1 mM DTT, 1 mM MgCl_2_) including restriction enzymes, and then incubated for 10 °C for 1 hour. The digestion buffer was recovered as supernatants (sup), and the magnetic beads were washed with elution buffer as beads fractions.

### Gel mobility shift assay

The gel shift assay of cssDNA was carried out in agarose gel electrophoresis with TAE buffer. cssDNA of pUC119 (100 ng) in HKD buffer including 1 mM MgCl_2_ was incubated at 4 °C for 10 min. *E .coli* SSB was purchased from Bio Academia. The agarose gels were stained by SYBRGreen II (Takara Bio Inc.). Images of gels were captured by using LuminoGraph (ATTO) and band intensities were analyzed by using ImageJ 1.48v.

## Additional Information

**How to cite this article**: Niki, H. and Yano, K. *In vitro* topological loading of bacterial condensin MukB on DNA, preferentially single-stranded DNA rather than double-stranded DNA. *Sci. Rep*. **6**, 29469; doi: 10.1038/srep29469 (2016).

## Supplementary Material

Supplementary Information
