# Supplementary material for: In vitro topological loading of bacterial condensin MukB on DNA, preferentially single-stranded DNA rather than double-stranded DNA
Source: Sci Rep. 2016 Jul 8;6:29469. doi: 10.1038/srep29469 (PMC4937444; doi:10.1038/srep29469)
Supplement: Supplementary Information [file srep29469-s1.pdf]

Supplementary Information

In vitro topological loading of bacterial condensin MukB on DNA, preferentially single-stranded DNA rather than double-stranded DNA

Hironori Niki[1, 2, *] and Koichi Yano[1]

[1] Microbial Genetics Laboratory, Genetic Strains Research Center,

National Institute of Genetics

1111, Yata, Mishima, Shizuoka, Japan. 411-8540

[2] Department of Genetics, SOKENDAI (The Graduate University for Advanced Studies)

1111, Yata, Mishima, Shizuoka, Japan. 411-8540

*To whom correspondence should be addressed.

Purification of histidine-tagged Muk proteins

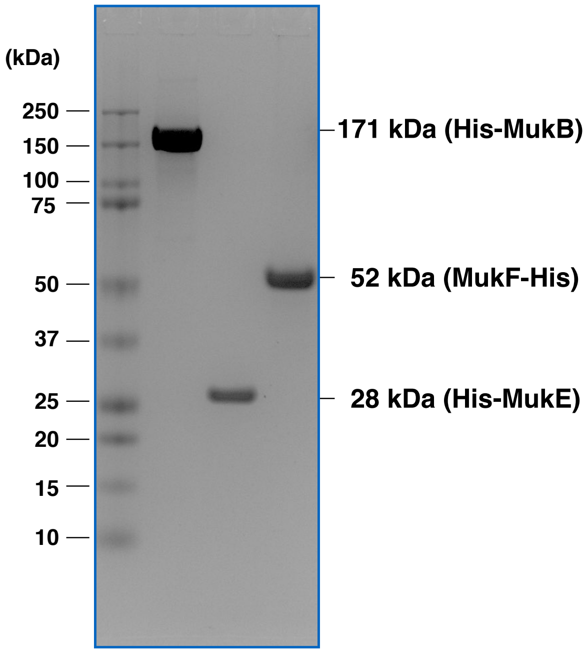

Supplementary Fig. S1

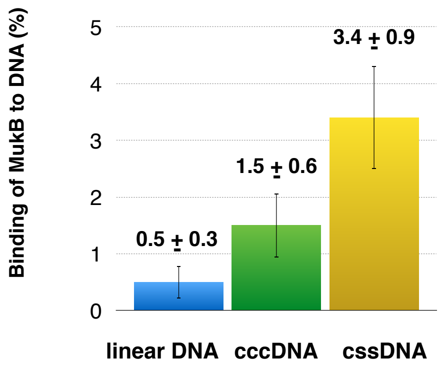

Supplementary Fig. S2

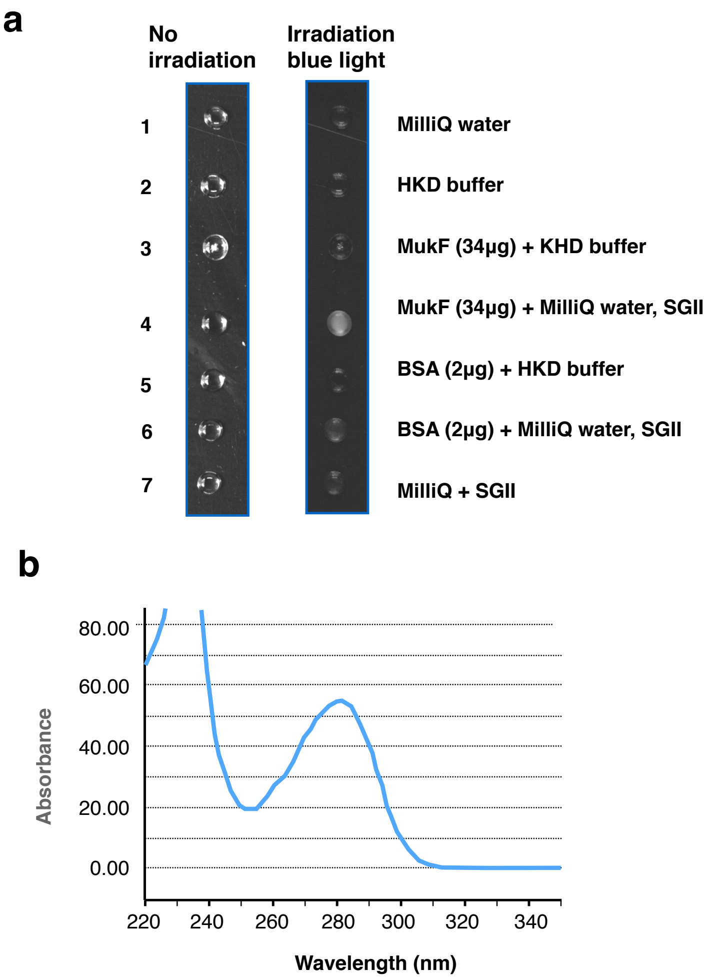

Supplementary Fig. S3

Extended Data Fig. S1 SDS polyacrylamide gel electrophoresis

Purified histidine-tagged proteins, MukB, MukE, and MukF were analyzed by using SDS polyacrylamide gel electrophoresis. Each lane was loaded on 36.5 pmol of the purified histidine-tagged proteins. Proteins in the gel were stained by Coomassie Brilliant Blue R-250.

Supplementary Fig. S2 In vitro loading of MukB onto DNA

Quantification of retrieved DNA by the MU assay. The means and standard deviations were calculated from three independent experiments. The lot number of histidine-tagged MukB used in this experiment was different from that of the purified protein used in the experiments of Fig. 3b.

Extended Data Fig. S3 Fluorescence and absorbance spectrum of MukF

a, The fluorescence of solutions (10 μl) was detected. **b,** The absorbance spectrum of a solution including MukF (34 μg) was measured by using a spectrophotometer (NanoDrop ND-1000).
